# It's Not the Genes OR the Environment, It's the Genes AND the Environment!

**DOI:** 10.1161/JAHA.121.022422

**Published:** 2021-09-02

**Authors:** Redford B. Williams

**Affiliations:** ^1^ Departments of Psychiatry and Medicine Duke University School of Medicine Durham NC

**Keywords:** Editorials, candidate genes, depression, environment, genetic epidemiology, prevention, Etiology, Mechanisms, Pathophysiology, Translational Studies

In this issue of the *Journal of the American Heart Association (JAHA)*, Jones et al used a Mendelian randomization approach to document protective effects of higher education levels in terms of reduced depression, anxiety, and cardiovascular disease (CVD) in a sample of over 300 000 participants in the UK Biobank study.^1^ They also found that Mendelian randomization analyses provide evidence that the reduced depression associated with higher education level accounts for 5.8% of the negative association between education and CVD and that 29% of this protective effect is mediated by reduced smoking. These findings are likely valid and have implications for interventions to reduce CVD in people with low education. There are several concerns, however, that need to be taken into account in evaluating this and similar studies.

The major concern is that all the gene loci identified by the genome‐wide association study analyses to be used in the Mendelian randomization analyses for association with the outcomes of interest in the Jones et al study do not take into account the fact that environmental factors, for example, low education, can moderate the influence of gene variants on the expression of a broad range of phenotypes, including those evaluated in the current study.

The pathway from educational attainment to CVD via depression can be a lot more complicated than that shown in figure 5 of the Jones et al study. The gene × environment (G×E) model^2^ shown in Figure 1 indicates, for example, that there are many pathways whereby environmental factors like education level can influence the expression of a broad range of phenotypes. This G×E model indicates, moreover, that genetic factors can moderate the effects of the environmental factor on the ultimate outcome of CVD at several points along the way.

**Figure 1:**
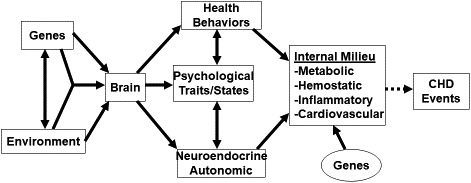
Causal model whereby genes and environment affect brain systems responsible for expression of health behaviors, psychological traits and states, and neuroendocrine and autonomic functions that influence components of the body's internal milieu in ways that, over time, lead to the development of coronary heart disease. Reproduced with permission from the Annual Review of Clinical Psychology, Volume 4 © 2008 by Annual Reviews, http://www.annualreviews.org ^2^. CHD indicates coronary heart disease.

A study by Singh et al^3^ provides an example of genetic moderation of such a pathway from an environmental factor—chronic psychosocial stress—to a measure of CVD—common carotid intimal‐media thickness. This study used a validated stress measure in MESA (Multi‐Ethnic Study of Atherosclerosis) to conduct a genome‐wide association study×stress analysis. There were 5 single nucleotide polymorphisms (SNPs) in the early B‐cell factor 1 gene for which the SNP×stress interaction *P* values were genome‐wide significant. In SNP‐only analyses the *P* values for these SNPs were not significant at the conventional genome‐wide significance level. Also, as shown in Figure 2, a structural equation path analysis found that the path from chronic psychosocial stress to common carotid intimal‐media thickness via hip circumference and fasting glucose was significant and larger in White MESA participants with the early B‐cell factor 1 rs4704963 CT/CC genotype than the same path that was nonsignificant in those with the TT genotype. There was also a stronger path from stress to glucose to common carotid intimal‐media thickness in the CT/CC group. It is noteworthy that when the same G×E genome‐wide association study was done in Chinese American, Black, and Hispanic MESA participants, there was no replication of any SNP across these groups for the gene by stress interaction.

**Figure 2:**
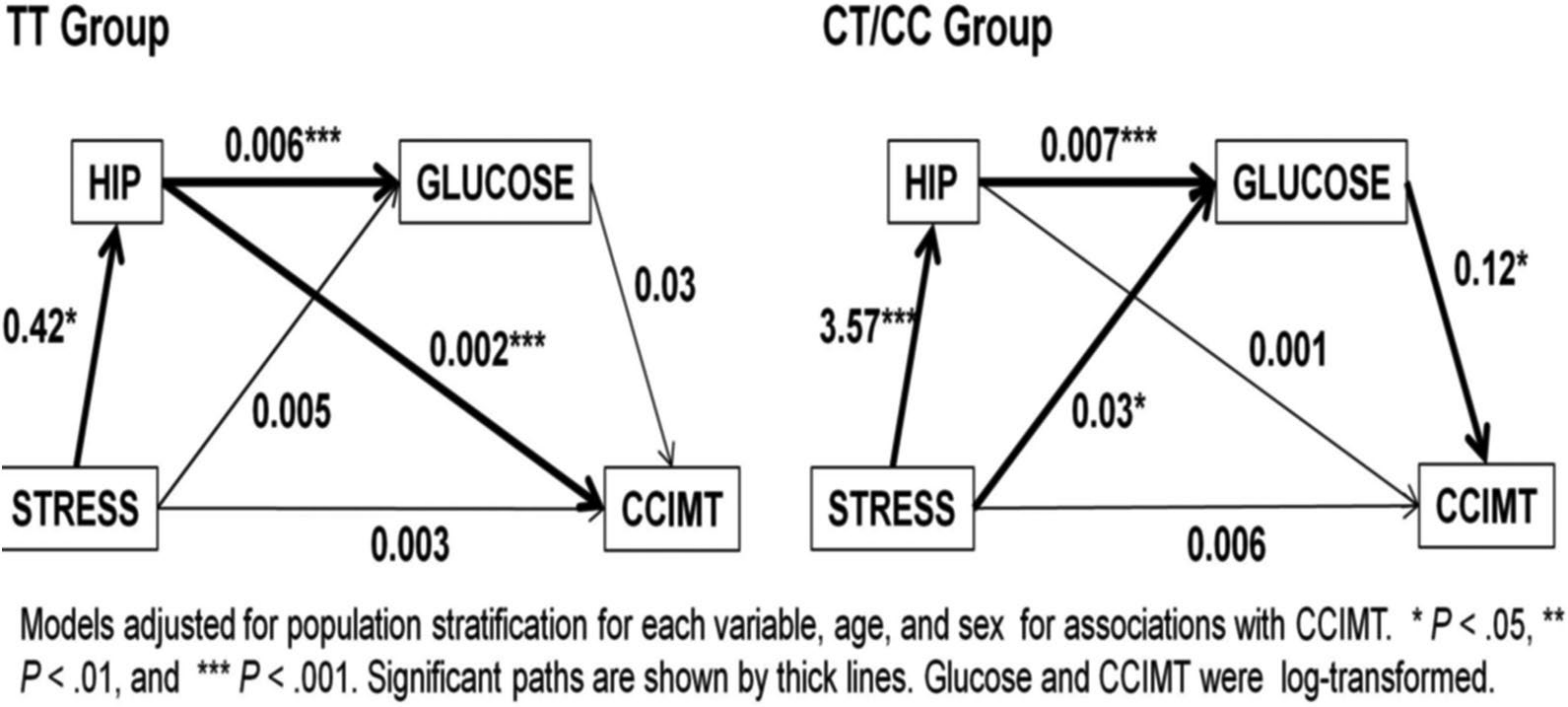
Structural equation path models of proposed direct and indirect effects among chronic psychosocial stress, hip circumference, fasting glucose, and common carotid intimal–medial thickness (CCIMT) for the 2 genotype groups (TT and CT/CC) of Early B‐cell Factor 1 (EBF1) single nucleotide polymorphism (SNP) rs4704963:T4C in White MESA participants. Values represent unstandardized path (slope) coefficients. Data were adjusted for ancestry stratification in a preliminary step. Not depicted in the figure are age and sex, which were included as covariates, adjusting all paths leading to hip circumference, glucose, and CCIMT. Glucose and CCIMT were transformed using the natural logarithm. (Adapted from Singh et al^3^; https://www.nature.com/articles/ejhg2014189).

A study by Williams et al^4^ provides evidence that both race and sex moderate the association of a functional insertion/deletion polymorphism (5HTTLPR) in the promoter region of the serotonin transporter gene with cerebrospinal fluid (CSF) levels of the major serotonin metabolite 5HIAA. In White people the 5HTTLPR long/long genotype is associated with higher levels of CSF 5HIAA, but in Black people the short/short (S/S) genotype is associated with higher CSF 5HIAA levels. A similar interaction was observed for gene by sex. In women CSF 5HIAA levels were higher in those with the S/S genotype, whereas in men it was those with the long/long genotype who had higher CSF 5HIAA levels. Not surprisingly, looking at both race and sex simultaneously, Black women had CSF 5HIAA levels that were nearly 3 times higher than those in White men.

It is known that central nervous system serotonin levels are involved in the regulation of a broad range of phenotypes. It is important to note that considerable evidence suggests that the influence of genes related to serotonin function on expression of a broad range of phenotypes can also be moderated by both race and sex. In a study of population differences in associations of 5HTTLPR genotypes with blood pressure and hypertension prevalence, Williams et al^5^ found that in White people those carrying the more active 5HTTLPR L' (takes into account rs25531 A‐G SNP effect on activity of L allele) had higher blood pressure and severity of hypertension stage, whereas in Black, Asian/Pacific Islander, and Native American people the S' allele was associated with higher blood pressure levels and severity of hypertension stage.

Following up the earlier observation that sex moderates the association between 5HTTLPR genotype and CSF 5HIAA,^4^ Brummett et al^6^ evaluated sex as a moderator of the influence of stress on symptoms of depression in 2 independent samples. In both samples the stress group (caregiver of a patient with Alzheimer's disease or father's low education level)×5HTTLPR×sex interaction was significant. For women the S allele was associated with higher depression levels in the caregivers (sample 1) and those whose father had a low education level (sample 2). In contrast among men it was those in the high stress group with the L allele in both samples who had higher depression levels.

There are also several studies that have found gene variants to moderate the influence of stress on expression of various phenotypes. In a study of sleep quality in 142 caregivers of a spouse or parent with dementia and 146 noncaregiver controls, Brummett et al^7^ found a significant caregiver×5HTTLPR interaction, such that the S allele was associated with poor sleep quality in caregivers compared with controls. In contrast to caregivers, among whom those with the S/S genotype had the highest poor sleep quality, controls with the long/short and S/S genotypes had better sleep quality than those with the long/long genotype. In other words, the same S allele that was associated with poorer sleep quality in caregivers was associated with better sleep quality in controls.

In a study more explicitly documenting this opposite effect of a genetic variant upon a phenotype in high versus low stress groups, Belsky et al^8^ found that in female caregivers with the 5HTTLPR S/S genotype their mean Center for Epidemiologic Studies Depression scale score was in the clinical range—18. In marked contrast, among the controls those with the S/S genotype had a very low score of 4.

This opposite effect of the same genotype—bad in the high stress group versus good in the low stress group—has been documented in 2 additional studies. Kring et al^9^ found that among caregivers of a patient with dementia those with the T/T genotype of apoE (apolipoprotein E) rs439401 had higher levels of triglyceride than C allele carriers. In marked contrast, among the controls those with the T/T genotype had much lower levels of triglyceride. A similar pattern was found for TOMM40 rs157580 by Jiang et al^10^ in a study of caregivers and controls. Among caregivers, those carrying the G allele had higher triglyceride levels than those with the A/A genotype, whereas among controls those carrying the G allele had lower levels than those with the A/A genotype.

A major implication of the studies reviewed is that interventions to reduce the health‐damaging effects of particular stressors and/or genotypes need to take into account the possibility that among those exposed to a stressor known to increase risk of disease, it may be those with a specific genotype who are most harmed and who should be targeted for interventions that will reduce the harm done by the bad G×E interaction. Moreover, the genotype that increases disease risk among those exposed to stress may differ as a function of race or sex. In the Brummett et al study^6^ of 5HTTLPR×stress effects on depression levels, for example, any intervention aimed at reducing the adverse impact of 5HTTLPR genotype on depression levels in those exposed to high stress would be more likely to be successful if it targeted men with the 5HTTLPR long/long genotype and women with the S/S genotype.

Even if we can use this research documenting G×E effects on disease risk to identify people most likely to benefit from interventions aimed at reducing those effects, the question remains, what sorts of interventions are most likely to benefit those identified as being at highest risk owing to psychosocial indices of stress?

One place to start addressing this question is the large‐scale, multicenter, National Heart, Lung, and Blood Institute‐sponsored ENRICHD (Enhancing Recovery in Coronary Heart Disease Patients) randomized trial that evaluated individual cognitive behavior therapy plus group therapy when feasible to reduce morbidity and mortality in patients post‐myocardial infarction who met criteria for depression or low perceived social support. As reported in the final report of the ENRICHD trial major outcomes,^11^ "The intervention trial did not increase event‐free survival." In a follow‐up study evaluating the impact of the group intervention component of the ENRICHD trial among the nonrandom 356 patients who were able to participate in the group intervention, Saab et al^12^ conducted analyses correcting for differential survival among comparison groups and found that group plus individual therapy was associated with a 33% reduction (*P*=0.01) in medical outcomes compared with patients randomized to usual care. No significant effect on event‐free survival was found in the group who received only individual therapy. In a multivariate‐adjusted model the group training benefit was reduced to 23% (*P*=0.11).

The ENRICHD group intervention was presented in 12 two‐hour sessions and provided training in evaluation of thoughts, feelings, and behaviors in distressing situations; management of negative thinking and emotions; problem‐solving and assertion; communication and social support; personal values; life goals; and maintenance of behavior change. Each session included homework review, sharing among group members, practice with training for each skill, and homework assignment.

Support for the clinical benefit of such interventions is provided by 2 randomized clinical trials of group‐based cognitive‐behavioral stress management training that reduced morbidity and mortality among patients with coronary heart disease in Sweden^13^, ^14^ that are cited by Jones et al.^1^

In conclusion, the Jones et al^1^ study provides encouraging evidence that we will ultimately be able to identify stressors that increase risk of CVD and the mediators in the pathway(s) from stress to disease. As reviewed in the foregoing, however, success in understanding how stress leads to disease and using that knowledge to reduce disease events will require that we continue the work that will enable us to understand the role of genetic variants in moderating the impact of the stressors on mediators in different race and sex groups. It will also require using that knowledge and the knowledge gained in clinical trials like those reviewed in the foregoing to develop cognitive‐behavioral stress management training interventions that will reduce the impact of stress on the mediators in the pathways from it to disease.

It's not the genes OR the environment, it's how the genes AND the environment influence the development of disease that we will ultimately need to understand in order to develop and implement the most effective means of preventing environmental stressors from leading to the development of disease.

## Sources of Funding

The author is supported in part by institutional research grant support from National Institutes of Health (P01 HL036587)

## References

[1] Jones D , Wootton R , Gill D , Carter A , Gunnell D , Munafo M , Sallis H . Mental health as a mediator of the association between educational inequality and cardiovascular disease: a Mendelian randomisation study. J Am Heart Assoc. 2021;10:e019340. DOI: 10.1161/JAHA.120.019340. 34472355 PMC8649303

[2] Williams RB . Psychosocial and biobehavioral factors and their interplay in coronary heart disease. Annu Rev Clin Psychol. 2008;4:349–365. DOI: 10.1146/annurev.clinpsy.4.022007.141237. 17716037

[3] Singh A , Babyak MA , Nolan DK , Brummett BH , Jiang R , Siegler IC , Kraus WE , Shah SH , Williams RB , Hauser ER . Gene by stress genome‐wide interaction analysis and path analysis identify EBF1 as a cardiovascular and metabolic risk gene. Eur J Hum Genet. 2015;23:854–862. DOI: 10.1038/ejhg.2014.189. 25271088 PMC4795045

[4] Williams RB , Marchuk DA , Gadde KM , Barefoot JC , Grichnik K , Helms MJ , Kuhn CM , Lewis JG , Schanberg SM , Stafford‐Smith M , et al. Serotonin‐related gene polymorphisms and central nervous system serotonin function. Neuropsychopharmacology. 2003;28:533–541. DOI: 10.1038/sj.npp.1300054. 12629534

[5] Williams RB , Bishop GD , Haberstick BC , Smolen A , Brummett BH , Siegler IC , Babyak MA , Zhang X , Tai ES , Lee J‐M , et al. Population differences in associations of serotonin transporter promoter polymorphism (5HTTLPR) di‐ and triallelic genotypes with blood pressure and hypertension prevalence. Am Heart J. 2017;185:110–122. DOI: 10.1016/j.ahj.2016.12.013. 28267464 PMC5473420

[6] Brummett BH , Boyle SH , Siegler IC , Kuhn CM , Ashley‐Koch A , Jonassaint CR , Züchner S , Collins A , Williams RB . Effects of environmental stress and gender on associations among symptoms of depression and the serotonin transporter gene linked polymorphic region (5‐HTTLPR). Behav Genet. 2008;38:34–43. DOI: 10.1007/s10519-007-9172-1. 17955359 PMC2777886

[7] Brummett BH , Krystal AD , Ashley‐Koch A , Kuhn C , Züchner S , Siegler IC , Barefoot JC , Ballard EL , Gwyther LP , Williams RB . Sleep quality varies as a function of 5‐HTTLPR genotype and stress. Psychosom Med. 2007;69:621–624. DOI: 10.1097/PSY.0b013e31814b8de6. 17766685 PMC2758820

[8] Belsky J , Jonassaint C , Pluess M , Stanton M , Brummett B , Williams R . Vulnerability genes or plasticity genes? Mol Psychiatry. 2009;14:746–754. DOI: 10.1038/mp.2009.44. 19455150 PMC2834322

[9] Kring SI , Brummett BH , Barefoot JC , Garrett ME , Ashley‐Koch AE , Boyle SH , Siegler IC , Sorensen TIA , Williams RB . Impact of psychological stress on the associations between APOE variants and metabolic traits: findings in an American sample of caregivers and controls. Psychosom Med. 2010;72:427–433. 20467002 10.1097/PSY.0b013e3181de30ad PMC3625667

[10] Jiang R , Brummett BH , Hauser ER , Babyak MA , Siegler IC , Singh A , Astrup A , Pedersen O , Hansen T , Holst C , et al. Chronic family stress moderates the association between a TOMM40 variant and triglyceride levels in two independent Caucasian samples. Biol Psychol. 2013;93:184–189. DOI: 10.1016/j.biopsycho.2013.02.006. 23435269 PMC3739426

[11] Berkman LF , Blumenthal J , Burg M , Carney RM , Catellier D , Cowan MJ , Czajkowski SM , DeBusk R , Hosking J , Jaffe A , et al. Effects of treating depression and low perceived social support on clinical events after myocardial infarction: the Enhancing Recovery in Coronary Heart Disease Patients (ENRICHD) Randomized Trial. JAMA. 2003;289:3106–3116. 12813116 10.1001/jama.289.23.3106

[12] Saab PG , Bang H , Williams RB , Powell LH , Schneiderman N , Thoresen C , Burg M , Keefe F ; ENRICHD Investigators . The impact of cognitive behavioral group training on event‐free survival in patients with myocardial infarction: the ENRICHD experience. J Psychosom Res. 2009;67:45–56. DOI: 10.1016/j.jpsychores.2009.01.015. 19539818 PMC2774492

[13] Gulliksson M , Burell G , Vessby B , Lundin L , Toss H , Svärdsudd K . Randomized controlled trial of cognitive behavioral therapy vs standard treatment to prevent recurrent cardiovascular events in patients with coronary heart disease: Secondary Prevention in Uppsala Primary Health Care project (SUPRIM). Arch Intern Med. 2011;171:134–140. DOI: 10.1001/archinternmed.2010.510. 21263103

[14] Orth‐Gomér K , Schneiderman N , Wang HX , Walldin C , Blom M , Jernberg T . Stress reduction prolongs life in women with coronary disease: the Stockholm Women's Intervention Trial for Coronary Heart Disease (SWITCHD). Circ Cardiovasc Qual Outcomes. 2009;2:25–32. DOI: 10.1161/CIRCOUTCOMES.108.812859. 20031809

